# Prevalence of *Chlamydia trachomatis* and *Neisseria gonorrhoeae* infections among pregnant women and eye colonization of their neonates at birth time, Shiraz, Southern Iran

**DOI:** 10.1186/s12879-018-3382-4

**Published:** 2018-09-24

**Authors:** Bahman Pourabbas, Zahra Rezaei, Jalal Mardaneh, Mozhgan Shahian, Abdolvahab Alborzi

**Affiliations:** 1 Professor Alborzi Clinical Microbiology Research Center, Shiraz University of Medical Sciences, Shiraz, Iran; 2 Departments of Microbiology, School of Medicine, Gonabad University of Medical Sciences, Gonabad, Iran

**Keywords:** *C. Trachomatis*, *N. Gonorrhoeae*, Pregnant women, Vertical transmission, Eye colonization

## Abstract

**Background:**

*Chlamydia trachomatis* and *Neisseria gonorrhoeae* are the two common transmissible pathogens from pregnant women to their neonates. Given the lack of routine screening and treatment of pregnant women in some areas, the possibility of transmission rises. This study seeks to determine the prevalence of *C. trachomatis* and *N. gonorrhoeae* in the pregnant women with no clinical symptoms and the vertical transmission rate to their neonates.

**Methods:**

The study was conducted on endocervical and eye swab samples of 239 pregnant women and their neonates. Identification was based on PCR method.

**Results:**

The prevalence rates of *C.trachomatis* in women and neonates were 37/239 (15.5%) and 28/239 (11.7%), and for *N. gonorrhoeae* 3/239 (1.3%), 1/239 (0.4%), respectively. The vertical transmission rates to the neonates were 28/37(75.6%) for *C. trachomatis* and 1/3 for *N. gonorrhoeae*.

**Conclusions:**

In the areas with a high prevalence of chlamydial or gonococcal infections, and in the absence of screening and treatment of the pregnant women, ocular prophylaxis with antibiotics is suggested as a part of routine neonatal care program for the prevention of chlamydial and gonococcal ophthalmia.

## Background

*C. trachomatis* and *N. gonorrhoeae* are the common prevalent sexually transmitted bacteria capable of infecting men, women and neonates worldwide, especially in developing nations [1, 2]. The estimated global prevalence in 2012 was 131 million cases of *C. trachomatis* and 78 million cases of *N. gonorrhoeae* in adults between the age of 15 and 49 years [2]. The annual estimation indicates different rates of infection in various parts of the world, ranging between 1.9–30.6% in pregnant women [2] and 1.6–18% in the neonates [3, 4] for *C. trachomatis* and for *N. gonorrhoeae* 0.08–7% [2] and 0.06–0.4% [3, 5], respectively. In Iran, the results of a meta-analysis showed that the pooled prevalence rates of *C. trachomatis* in males and females were 10.9% (95% CI, range: 7.6–15.4%) and 12.3% (95% CI, range: 10.6–14.2%), respectively [6]. Also, studies reported that prevalence of *C. trachomatis* in pregnant women was 2.2% to 14.79% [7–9]. As for *N. gonorrhoeae,* studies in Iran showed that its prevalence is 0 to 2.4% in people at low risk without symptoms [10]. One study on pregnant women revealed that 1.18% samples were positive for *N. gonorrhoeae* by PCR [11]. Infection with these organisms can cause a range of serious problems in mothers and newborns and can be asymptomatic in a significant proportion of the affected individuals [12, 13]. *C. trachomatis *is the most frequent infectious agent accounting for 18% to 50% of all neonatal conjunctivitis and 3% to 20% of infantile pneumonia [14, 15]. About half of the neonates born from infected mothers with *N. gonorrhoeae* will develop neonatal conjunctivitis [16]. Untreated gonococcal conjunctivitis may lead to corneal scarring and blindness, whereas the risk of severe ocular damage is low with chlamydial infection which can cause purulent mucous, edema on the eyelids, papillary conjunctivitis, and the formation of pseudomembranes [16]. The transmission of these two organisms is usually through direct contact of the neonates with infected vagina at the time of delivery during vaginal birth but rarely occurs during Caesarean section [17].

Prenatal screening and treatment of pregnant women are highly recommended, but in Iran, the routine prenatal and prophylactic care to prevent these diseases in newborns is lacking, unlike some other countries in the developing world. The aim of this study is to determine the prevalence of *C. trachomatis* and *N. gonorrhoeae* in pregnant women admitted to the hospitals before delivery and respective vertical transmission rates to the neonates, using PCR method.

## Methods

A total of 239 pregnant women referred to Hafez and Zeinabiye hospitals for delivery were enrolled in the study during 3 months. Over 50% of all the deliveries in Shiraz are performed in these two hospitals, with more than 60% vaginal delivery. Pregnant women from different regions and of various socioeconomic statuses are admitted. This study includes the women with vaginal delivery with no history of antibiotics prepartum and their neonates had not received prophylactic ophthalmic antibiotics, either. Endocervical and eye swab samples were obtained from the mothers and their neonates, respectively. The former were taken by a sterile cotton swab from endocervix before delivery and the latter by the scraping of upper lid conjunctiva, using a sterile cotton swab immediately after delivery. Both swabs were placed in separate tubes containing 500 μl of sucrose phosphate transport medium (8 mM KH_2_PO_4_, 12 mM K_2_HPO_4_ and 0.2 mM sucrose) supplemented with antibiotics (amphotricin B 2.5 μg/ml, streptomycin 50 μg/ml and vancomycin 100 μg/ml) and transported to Professor Alborzi Clinical Microbiology Research Center, the same day for DNA extraction. The sample tubes were vortexed, swabs removed, and then, 250 μl of the sample transferred to a 1.5 ml tube containing 250 μl 10 mM Tris-HCl, pH 8.0 and 1 mM EDTA (TE). Each sample supplemented with 4 μl proteinase K (10 μg/ml) and 250 μl TNNT buffer (0.5% Tween 20, 0.5% Nonidet P-40, 10 mM NaOH, 10 mM Tris,pH 7.2), was incubated at 56 °C overnight and then, DNA extraction was performed using phenol chloroform method and finally, DNA resolved in 50 μl TE buffer and stored at -20 °C. Detection of *N. gonorrhoeae* and *C. trachomatis* was through two single-plex conventional PCR kits (Sacace Biothecnologies co. Italy) with specific primers for cryptic plasmid and amino acyltransferase gene. Five μl of DNA was amplified in a epgradiant thermocycler (eppendorf, Germany) using the following protocol: an initial denaturation (94 °C, 5 min), followed by 42 cycles of denaturation (94 °C, 1 min), annealing (54 °C, 1 min) and extension (72 °C, 1 min), and a single final extension of 1 min at 72 °C for both *C. trachomatis* and *N. gonorrhoeae*, according to the manufacturer’s instructions. These kits allow detecting DNA in 100% of the tests with a sensitivity of not less than 1000 copies/ml and a specificity of 100%.

The collected data were entered into SPSS, version 18. The statistical relationship between the demographic data and infection was assessed using the chi-square and t-test.

## Results

### Subjects

Of the 239 studied women with vaginal delivery (mean age 27.7; range: 18–45 years), 31.7% were illiterate or had primary education, 65% with secondary education, and 3.3% with university degrees. In total, 98% of them were housewives. The median gestational age was 39 weeks with a range of 32 to 44 weeks. Of the 239 studied neonates, 55% were male and 45% were female.

### Diagnosis of *C. trachomatis* and *N. gonorrhoeae* infection in pregnant women

From 239 endocervical swabs, DNAs of *C. trachomatis* and *N. gonorrhoeae* were detected in 37 (15.4%) and 3 (1.3%), respectively. No case of co-infection with *N. gonorrhoeae* and *C. trachomatis* was observed.

No significant association was found between *C.trachomatis*, *N. gonorrhoeae* prevalence and age and educational status in the pregnant women in this study (*P*-value > 0.05).

### Diagnosis of *C. trachomatis* and *N. gonorrhoeae* infection in neonates

Of the 239 samples from neonatal eyes, DNAs of *C. trachomatis* and *N. gonorrhoeae* were detected in 28 (11.7%) and 1 (0.4%), respectively. Transmission rates of infection in the neonates for *N. gonorrhoeae* and *C. trachomatis*, born to the infected mothers, were 28/37 (75.6%) and 1/3, respectively. No case of co-infection with *N. gonorrhoeae* and *C. trachomatis* was detected in the neonates, as was the case with mothers.

## Discussion

*C. trachomatis* and *N. gonorrhoeae* are the most common causes of sexually transmitted infections leading to serious conditions in pregnant women and their neonates. Prior to this study, limited data existed on *C. trachomatis* and *N. gonorrhoeae* prevalence among the Iranian pregnant women with no clinical symptoms and their neonates. Therefore, the present study provides documented data for the first time in Iran on the vertical transmission rates of these two organisms in the neonates. A PCR assay which is more sensitive and specific, compared to other methods such as culture as gold standard, was used [18, 19].

The results revealed that 37 (15.5%) pregnant women were infected with C*. trachomatis.* Previous studies showed that the frequency of *C. trachomatis* among women varies in different countries, and even within the same country.

Previous studies have reported that the frequency of *C. trachomatis* infection among pregnant women is 2.5 to 4.74% in the United States [20, 21], 1.9 to 14.9% in Asia [2], 1.6 to 16.4% in Europe [2, 22], 5.2 to 18.6% in Africa [2], and 10.9 to 30.6% in Oceania [2]. Differences in frequency could be explained by sociodemographic factors; different sensitivities of the diagnostic methods used and specimen types collected. Unlike in some previous studies, no association was found between *C.trachomatis* prevalence and age and educational status in the pregnant women in the present study [23, 24].

The prevalence of eye colonization with *C. trachomatis* in the neonates was found to be 11.7% in this study, which varies in different studies, ranging between 1.6 to 18% [3, 4]. This difference is probably due to the different sensitivity of the methods used in such studies and varying prevalence of infections among mothers throughout the world.

The vertical transmission rate of *C. trachomatis* was 75.6% which is in agreement with reported rates in other studies [3, 4] but in contrast to a study by Jilian et al., which reported a lower rate of vertical transmission [25]. The discrepancy in these two findings may be due to different target populations [25].

Consistent with other studies*, N. gonorrhoeae* had a low prevalence of 1.3% in the pregnant women [26, 27]. Similar to *C. trachomatis*, there was no significant relationship between the prevalence of *N. gonorrhoeae* and the mothers’ educational status and age. The prevalence of eye infection and rate of vertical transmission with *N. gonorrhoeae* in the neonates were 0.4% and 1/3 respectively, consistent with some other studies [3, 28]. No concomitant *C. trachomatis* and *N. gonorrhoeae* infection was detected in mothers or neonates while all positive neonates were born to the infected mothers.

Studies show that ocular prophylaxis in neonates was effective against gonoccocal eye infection, whereas it may not be the case for chlamydial conjunctivitis [29, 30]. Neither can it prohibit colonization or infection at other sites like nasopharynx, rectum, and vagina; conversely, it may increase antimicrobial resistance and cause some level of toxicity [31, 32]. Overall, ocular prophylaxis may help to prevent chlamydial neonatal conjunctivitis, but not that much to help to prevent gonococcal neonatal conjunctivitis [29].

In Iran, prenatal screening and treatment is not a part of the routine program for mothers care and consequently there is a chance of transmitted *C. trachomatis* and *N. gonorrhoeae* as a common cause of neonatal conjunctivitis.

## Conclusion

Prenatal screening and treatment of pregnant women have been demonstrated to be very effective for the prevention of neonatal chlamydial and gonococcal infections. Given the above mentioned findings, we can conclude that in the absence of a program for screening and treatment of pregnant women and in areas with a considerable prevalence of such organisms, it is suggested that at least ocular prophylaxis with antibiotics be incorporated in the routine neonatal care program for the prevention of chlamydial and gonococcal ophthalmia.

## Abbreviations

*C. trachomatis*: *Chlamydia trachomatis*
*N. gonorrhoeae*: *Neisseria gonorrhoeae*

## References

[1] Darville T (2005). *Chlamydia trachomatis* infections in neonates and young children. Semin Pediatr Infect Dis.

[2] Newman L, Rowley J, Vander Hoorn S (2015). Global estimates of the prevalence and incidence of four curable sexually transmitted infections in 2012 based on systematic review and global reporting. PLoS One.

[3] Justel M, Alexandre I, Martínez P (2015). Vertical transmission of bacterial eye infections, Angola, 2011–2012. Emerg Infect Dis.

[4] Schachter J, Grossman M, Sweet RL (1986). Prospective study of perinatal transmission of chlamydia trachomatis. JAMA.

[5] Hammerschlag MR (2011). Chlamydial and gonococcal infections in infants and children. Clin Infect Dis.

[6] Ahmadi MH, Mirsalehian A, Bahador A (2015). Prevalence of genital chlamydia trachomatis in Iran: a systematic review and meta-analysis. Pathog Glob Health.

[7] Haghighi Hasanabad M, Mohammadzadeh M, Bahador A, Fazel N, Rakhshani H, Majnooni A (2011). Prevalence of Chlamydia trachomatis and Mycoplasma genitalium in. pregnant women of Sabzevar-Iran. Iran J Microbiol.

[8] Rashidi B, Chamani L, Haghollahi F (2009). Prevalence of *Chlamydia trachomatis* infection in fertile and infertile women; a molecular and serological study. J Reprod Infertil.

[9] Jahromi AS, Farjam MR, Mogharrab F, Amiryan M, Makiani MJ, Madani A (2010). *Chlamydia trachomatis* in women with full-term deliveries and women with abortion. Am J Infect Dis.

[10] Janghorban R, Azarkish F (2016). An overview on sexually transmitted infections in Iran. Int J Reprod Contracept Obstet Gynecol.

[11] Hassanzadeh P, Mardaneh J, Motamedifar M (2013). Conventional agar-based culture method, and nucleic acid amplification test (NAAT) of the cppB gene for detection of Neisseria gonorrhea in pregnant women Endocervical swab specimens. Iran Red Crescent Med J.

[12] Wager GP, Martin DH, Koutsky L (1980). Puerperal infectious morbidity: relationship to route of delivery and to antepartum chlamydia trachomatis infection. Am J Obstet Gynecol.

[13] Gencay M, Koskiniemi M, Ammaälaä P (2000). Chlamydia trachomatis seropositivity is associated both with stillbirth and preterm delivery. APMIS.

[14] Hammerschlag MR, Chandler JW, Alexander ER (1982). Longitudinal studies of chlamydial infection in the first year of life. Pediatr Infect Dis.

[15] American Academy of Pediatrics. Chlamydial infections. In: Pickering LK, Baker CJ, Long SS, eds. Red Book: 2006 Report of the Committee on Infectious Diseases. 27th ed. Elk Grove Village, Ill: Am Acad Pediatr. 2006;249–257.

[16] Zuppa AA, D’Andrea V, Catenazzi P (2011). Ophthalmia neonatorum: what kind of prophylaxis?. J Matern Fetal Neonatal Med.

[17] Bell TA, Stamm WE, Kuo CC (1994). Risk of perinatal transmission of chlamydia trachomatis by mode of delivery. J Inf Secur.

[18] Watson EJ, Templeton A, Russell I (2002). The accuracy and efficacy of screening tests for chlamydia trachomatis: a systematic review. J Med Microbiol.

[19] Centers for Disease Control and Prevention. Recommendations for the laboratory-based detection of Chlamydia trachomatis and Neisseria gonorrhoeae--2014. MMWR Recomm Rep. 2014;63(RR-02):1-19. PMC4047970 24622331

[20] Datta SD, Sternberg M, Johnson RE (2007). Gonorrhea and chlamydia in the United States among persons 14 to 39 years of age, 1999 to 2002. Ann Intern Med.

[21] Miller WC, Ford CA, Morris M, Handcock MS (2004). Prevalence of chlamydial and gonococcal infections among young adults in the United States. JAMA.

[22] Goulet V, de Barbeyrac B, Raherison S (2010). Prevalence of chlamydia trachomatis: results from the first national population-based survey in France. Sex Transm Infect.

[23] Romoren M, Sundby J, Velauthapillai M (2007). Chlamydia and gonorrhoea in pregnant Batswana women: time to discard the syndromic approach?. BMC Infect Dis.

[24] Harrison HR, Alexander ER, Weinstein L (1983). Cervical chlamydia trachomatis and mycoplasmal infections in pregnancy. Epidemiology and outcomes. JAMA.

[25] Yu J, Wu H, Li F (2009). Vertical transmission of chlamydia trachomatis in Chongqingn China. Curr Microbiol.

[26] Borges-Costa J, Matos C, Pereira F (2012). Sexually transmitted infections in pregnant adolescents: prevalence and association with maternal and foetal morbidity. J Eur Acad Dermatol Venereol.

[27] Panaretto KS, Lee HM, Mitchell MR (2006). Prevalence of sexually transmitted infections in pregnant urban aboriginal and Torres Strait islander women in northern Australia. Aust N Z J Obstet Gynaecol.

[28] Laga M, Plummer FA, Nzanze H (1986). Epidemiology of ophthalmia neonatorum in Kenya. Lancet.

[29] Darling EK, McDonald H (2010). A meta-analysis of the efficacy of ocular prophylactic agents used for the prevention of gonococcal and chlamydial ophthalmia neonatorum. J Midwifery Womens Health.

[30] Ramirez-Ortiz MA, Rodriguez-Almaraz M, Ochoa-Diazlopez H (2007). Randomised equivalency trial comparing 2.5% povidone-iodine drops and ophthalmic chloramphenicol for preventing neonatal conjunctivitis in a trachoma endemic area in southern Mexico. Br J Ophthalmol.

[31] Hammerschlag MR, Chandler JW, Alexander ER (1980). Erythromycin ointment for ocular prophylaxis of neonatal chlamydial infection. JAMA.

[32] Public Health Agency of Canada, National Microbiology Laboratory. National surveillance of antimicrobial susceptibilities of Neisseria gonorrhoeae. Annual Summary 2012: http://publications. gc.ca/collections/collection_2014/aspc-phac/HP57-3-2012-eng.pdf.

